# Basophil Activation Test as a Biomarker for Taxanes Anaphylaxis

**DOI:** 10.3389/falgy.2022.787749

**Published:** 2022-07-13

**Authors:** Lucila De Campos, Pedro Giavina-Bianchi, Shree Acharya, Donna-Marie Lynch, Jorge Kalil, Mariana C. Castells

**Affiliations:** ^1^Clinical Immunology and Allergy Division, University of São Paulo, São Paulo, Brazil; ^2^Division of Rheumatology, Immunology and Allergy, Department of Medicine, Brigham and Women's Hospital (BWH), Harvard Medical School, Boston, MA, United States

**Keywords:** basophil activation test (BAT), immediate hypersensitivity reaction, taxanes agents, anaphylaxis, chemotherapy

## Abstract

**Introduction:**

Taxanes are widely used chemotherapy agents, and their administration, despite premedication, is associated with hypersensitivity reactions (HR) in up to 9% of patients, 1% of which are severe. The mechanisms of these reactions are not fully understood. Finding biomarkers for early diagnosis and better understanding the underlying mechanisms of these reactions are key to defining the best treatment strategy for patients.

**Methods:**

The purpose of this study was to evaluate the effectiveness of the basophil activation test (BAT) to diagnose patients with anaphylactic reactions to taxanes. Patients with anaphylaxis to taxane compounds (*n* = 15) were assessed through clinical history, skin testing (when possible), and BAT. BAT was performed immediately before rapid drug desensitization or before skin testing using anti-CD123 conjugated (APC-Biolegend), anti-HLADR conjugated (FITC-Biolegend) to gate Basophils and anti-CD63 conjugated (PE-Biolegend), and anti-CD203c conjugated (BV-Biolegend) to assess CD203c and CD63 expression on basophils under taxane stimulation. BAT was also performed in eight healthy volunteers.

**Results:**

BAT was positive for CD203c in eight out of 15 patients and for CD63 in four out of 15 patients and in two out of eight controls. The sensitivity for CD203c was 53%, the specificity was 87%, and the area under the curve was 0.66 (*p* = 0.19%). For CD63, these rates were 33%, 87%, and 0.6 (*p* = 0.4). In a subgroup analysis of patients with positive skin tests (11 patients), CD203c was positive in six patients (sensitivity of 54.5% and specificity of 87.5%), and CD63 was positive in five patients (sensitivity of 45% and specificity of 75%).

**Conclusions:**

BAT as a diagnostic tool for immediate hypersensitivity reactions to taxanes may be relevant in patients with selected phenotypes and endotypes, especially those with severe reactions or when the diagnosis cannot be established by the skin test. Increased expression of CD203c was more frequent than of CD63 in patients with positive results, and the sensitivity of this biomarker was higher in patient sub-group with positive skin tests, i.e., patients with IgE-mediated endotypes.

## Introduction

Thanks to recent advances in cancer treatment, an increasing number of antineoplastic agents are available to patients, but the hypersensitivity reactions (HR) to these agents have also increased. In medical practice, most patients with HR to antineoplastic agents are classified as allergic and end up being deprived of first-line treatment for their disease, causing a negative impact on their quality of life and overall survival (1, 2). The accurate diagnosis of these patients by validated methods, as well as rapid drug desensitization (RDD) with the chemotherapeutic agent involved, could drastically change this perspective. Rapid drug desensitization (RDD) is a procedure that temporarily modifies a patient's immune response to a drug, generating clinical tolerance, allowing patients to receive fist line therapy for their underlying disease or cancer.

The incidence of HR to chemotherapeutic agents varies according to the agent used, being platinum and taxanes the most frequent (1, 2). Taxanes are an integral part of the treatment regimen administered to patients with various types of tumors, including breast, ovarian, prostate, and lung cancer, all highly prevalent (3). HR to taxanes (paclitaxel and docetaxel) occur mostly in the first or second exposure, minutes after the start of infusion, in 10% of patients receiving paclitaxel and 5% of patients receiving docetaxel (2, 4–7). The symptoms are compatible with those of an IgE-mediated immediate HR, often associated with atypical symptoms like back and abdominal pain, and 10% of these reactions can be severe (7). The pathophysiological mechanisms involved in these reactions are not yet fully understood, but three different mechanisms may be involved: complement activation with anaphylatoxin release, direct activation of mast cells and basophils, and, more recently put forth, evidence of IgE-mediated reactions (7).

The diagnosis of HR to chemotherapeutic agents is based on the clinical history (phenotype) and biomarkers (endotype), such as *in vivo* skin testing and validated *in vitro* tests, which are still scarce for this class of drugs (8). Immediate-reading skin testing for taxanes have high specificity (100%) but low sensitivity (8.7–24.6%). A greater number of positive skin tests has been observed in patients with more severe grade 3 reactions, indicating a greater likelihood of IgE-mediated reactions in these patients (9, 10).

The basophil activation test (BAT) has been evaluated as an *in vitro* diagnostic tool in different HR to drugs, including chemotherapeutic agents. BAT assesses the expression of cell-surface proteins related to basophil activation by flow cytometry after stimulation with the suspected allergen. The glycoprotein CD63 is found on the surface of the granules of basophils, and when the cell is activated by an allergen, the granules fuse to the cell membrane, promoting the expression of CD63 on the cell surface (11). Another molecule used to characterize basophilic activation is CD203c (12), which is expressed exclusively on the surface of basophils and mast cells (13). Unlike CD63, CD203c has basal constitutive expression in the basophil membrane, but it's expression increases significantly after activation.

In the study of immediate HR to chemotherapeutic agents, the basophil activation test was evaluated as an *in vitro* biomarker for the diagnosis of platinum compounds reactions, a scenario where serum specific IgE showed lower sensitivity than skin testing (14). BAT had a sensitivity of 73% and specificity of 100% (15). There is still no validation of *in vitro tests* for the diagnosis of HR to taxanes. Because there is evidence of an IgE-mediated mechanism in patients with positive skin tests and severe reactions, BAT may be a good *in vitro* approach for the diagnosis of these patients, as it could confirm the diagnosis and reduce the risk of severe reactions to chemotherapy. It can also help better understand the mechanisms besides those reactions.

We present here the largest series of patients with anaphylaxis to taxanes in whom CD63 and CD203c were evaluated by BAT as biomarkers of the immediate HR. Our data suggest that BAT, when positive, may be an additional diagnostic tool for patients with selected phenotypes and endotypes who have severe reactions and reactions with possible IgE-mediated mechanisms and to elucidate the mechanisms involved in the reactions. The confirmation of the etiological cause combined with the phenotype and grade of the reaction will drive treatment decisions, like the RDD protocol and premedication. The DFCI/BWH Desensitization Program for taxanes consists in a flexible 12-to-16 step protocol, accordingly to individual evaluation of the patient, which rendered mast cells unresponsive by delivering × 2 to × 2.5 doses of drug antigens at fixed time intervals starting at 1/1,000–1/100 dilutions of the final concentration (16).

## Methods

### Study Design

A prospective longitudinal study was conducted at Brigham and Women's Hospital and the Dana-Farber Cancer Institute affiliated with Harvard Medical School in Boston, Massachusetts, USA. The project was submitted to and approved by the research ethics committees Partners Institutional Review Board (protocols 13–288 and 2012P002275) and CAPPesq of the Hospital das Clínicas (Brazil Platform: CAAE: 22701913.6.0000.0068). Patients who agreed to participate signed an informed consent form and were included in the study. The study followed the Declaration of Helsinki and International Guidelines for Good Clinical Practice.

Cancer patients who had a documented history of anaphylaxis to taxanes chemotherapeutic agents from January 2019 to January 2020 were recruited. The severity of the reaction was assessed in patient records, according to Brown's classification as grade 1 (mild reactions with skin involvement), grade 2 (moderate reactions with cutaneous, cardiovascular, and/or respiratory impairment), or grade 3 (severe reactions with hypoxia, hypotension, and/or neurological impairment) (17). Only patients with grade 2–3 reactions were enrolled.

The *prick test* was performed as a routine test in the Allergy and Immunology Service for all patients that did not present any contra-indication for the procedure, before enrollment, following the Service protocol, with a drop of 0.4 mg/ml docetaxel or 1 mg/ml paclitaxel on the volar surface of the forearm, and the intradermal test was performed with a 0.03-ml injection in the anterior surface of the forearm at concentrations of 0.4 mg/ml docetaxel or 0.001 and 0.01 mg/ml paclitaxel. The tests were read after 20 min, and positive tests were those that triggered a wheal with a diameter at least 3 mm larger than that produced by the control (diluent). Histamine (10 mg/mL) was used as a positive control for the puncture test. Results of skin test were accessed retrospectively. Only patients with positive skin tests or patients unable to perform skin tests were included in the trial. Patients under chronic use of corticosteroids and patients with negative skin test were excluded from the study.

The BAT result was the primary outcome of this study, and the BAT results of the patients were compared to the results of the control group (healthy volunteers).

### Basophil Activation Test

Blood samples of the patients were obtained before desensitization and administration of prophylactic medications (antihistamines and corticosteroids) or on the day of immediate skin testing. Blood was collected in vacuum tubes containing heparin, and the assay was performed up to 6 h after collection. Two hundred microliters of whole blood was incubated with and without stimulus (100 μL paclitaxel or docetaxel) for 45 min at a temperature of 37°C, and at least two dilutions of each of active ingredient were tested. The dilutions were chosen based on the concentration used for the skin testing (paclitaxel: 1 mg/ml in the puncture test and 0.01 and 0.001 mg/ml in the intradermal test; docetaxel: 0.4 mg/ml in the puncture test and 0.04 mg/ml in the intradermal test) (9). The dilutions used for BAT were 0.1 mg/ml (1:10) and 0.01 mg/ml (1:100) for paclitaxel and 0.04 mg/ml and 0.004 mg/ml for docetaxel. For the results analysis the dilution showing the higher MFI values will be used.

At the end of the stimulation with the chemotherapeutic agent, the samples were kept at 4°C in the dark for 30 min, with the addition of 2.5 μl of the following antibodies: anti-CD123 conjugated with allophycocyanin (APC-Biolegend), anti-HLADR conjugated with fluorescein isothiocyanate (FITC-Biolegend), anti-CD63 conjugated with phycoerythrin (PE-Biolegend), and anti-CD203c conjugated with Brilliant Violet (BV-Biolegend). After 30 min of staining with the antibodies, the erythrocytes were removed by adding 4 ml of lysis solution (Lysis Solution—Biolegend), with a waiting time of 10 min and centrifugation for 5 min (Beckman GPR Centrifuge). Then, the cells were washed twice with 4 ml of FACS buffer solution, resuspended in 300 μl of the same solution, and analyzed in a flow cytometer (FACSCanto II, Becton Dickinson, San Jose, California, USA). The basophil population was delimited by the presence of CD123 expression (APC) and the absence of HLA DR expression (FITC). The minimum count of gated basophils was 50 and the median was 1,000. The CD63 and CD203c expression were analyzed within this population.

### Evaluation of the Results

The results of basophil activation are expressed as the median fluorescence intensity (MFI) of the cells. The results of the cytometry were analyzed using the program FlowJo version 9.0 (TreeStar, Ashland, Ore). As a positive control for the assay, a tube with 200 μl of whole blood, 100 μl of phosphate-buffered saline (PBS), and 3 μl of anti-IgE (Biolegend) was used. As a negative control, a tube with 200 μl of whole blood and 100 μl of PBS was used.

The CD-63 and CD203c increase were expressed as the ratio of the median fluorescence intensity (MFI) obtained with the drug to the MFI obtained with the negative control. This ratio is called stimulation index.

The sensitivity of BAT was calculated as the number of positive tests in the patients group divided by all the patients. The specificity was calculated as the number of negative tests correctly classified in the control group divided by all controls times 100.

### Statistical Analysis

Continuous variables are expressed as absolute value, relative percentage, or mean ± standard deviation (SD). Categorical variables are presented as raw number and percentage and were analyzed by the chi-squared test and Fisher's exact test. The cutoff point for BAT positivity (threshold) was calculated by drawing a receiver operating characteristic (ROC) curve. The ideal cutoff point for the test was where the sensitivity and specificity were as high as possible. The analyses were done in GraphPad Prism software (version 8), and a *p* < 0.05 was considered statistically significant.

## Results

### Characterization of the Samples

A total of 15 patients, 14 female and one male, with a mean age of 54 years old, who had documented anaphylactic reaction to taxanes underwent BAT. The general characterization of the patients and their initial reactions, in addition to the skin testing results and serum tryptase values during the initial reaction (when available), are shown in Table 1. The characteristics of the individuals of the control group are described in Table 2. The mean age of the control group was lower, 39 years old, and the group had 75% women, compared with 94% of the patient group. The time between initial reaction, skin test and BAT was >6 months in 14 patients and 11 months for 1 patient.

**Table 1:** General characteristics of patients and hypersensitivity reactions to taxanes (patients # 1 and # 8 were excluded due to initial reaction grade 1).

**Patient**	**Type of tumor**	**Sex**	**Age**	**Drug**	**Grade of initial reaction**	**Lifetime exposure**	**Symptoms**	**History of atopic disease/HR**	**Cutaneous tests**	**Tryptase**
Patient 2	Breast	F	40	Paclitaxel	3	First/second	Cutaneous + cardiovascular + swelling throat	NO	Contra-indicated/pregnant	NO
Patient 3	Gastric	F	70	Docetaxel	2	First/second	Flushing + respiratory	Asthma	ID + 1st dilution	NO
Patient 4	Uterine cancer	F	48	Paclitaxel	3	First	Flushing, respiratory, gastro-intestinal, cardiovascular with hypotension, desaturation and anaphylaxis	NO	ID + 1st dilution	NO
Patient 5	Ovarian	F	53	Paclitaxel	2	First	Flushing, respiratory, cardio-vascular, back pain, dizziness and urinary urgency (Central nervous system)	NO	ID + 1st dilution	2.9
Patient 6	Ovarian	F	59	Paclitaxel	3	First	Flushing, cardio-vascular, respiratory with desaturation—anaphylaxis	NO	ID + 2nd dilution	2.7
Patient 7	Ovarian	F	44	Paclitaxel	3	First	Cutaneous (hands and foot itching) + respiratory + gastro-intestinal	Asthma/drug reaction with sulfa and clindamycin	Caught and dyspnea during prick test—test interrupted	NO
Patient 9	Uterine cancer	F	72	Paclitaxel	3	First	Flushing, low back pain, cardio-vascular, central nervous system with syncope and hypotension	Anaphylaxis with nuts	ID + 1st dilution/negative Prick test to nuts	6.5
Patient 10	Ovarian	F	52	Paclitaxel	3	First	Cutaneous, respiratory and cardio-vascular with severe hypotension—Anaphylaxis, patient received epinephrine 3 times and developed delayed reaction (rash)	Iodinate contrast	Not realized—patient too weak	NO
Patient 11	Bladder	M	55	Paclitaxel	3	First	Cutaneous, central nervous system and respiratory with desaturation (anaphylaxis)	Asthma and urticaria	False negative, anti-histamines premedication	6.4, after 2 h 5.1
Patient 12	Breast	F	77	Paclitaxel	3	First	Flushing + respiratory with desaturation—anaphylaxis—epinephrine 1 x	NO	ID + 1st dilution	NO
Patient 13	Ovarian	F	61	Paclitaxel	3	First	Flushing, abdominal pain, cardiovascular, respiratory, desaturation—anaphylaxis	Allergic Rhinitis (cat)	ID + 1st dilution	NO
Patient 14	Ovarian	F	66	Paclitaxel	3	First	Low back pain, respiratory and cardiovascular (tachycardia)	Allergic Rhinitis (cat/dog)/sulpha	ID + 1st dilution	NO
Patient 15	Breast	F	31	Paclitaxel	3	First	Cutaneous and respiratory	Urticaria (sulpha and penicillin)	False positive, dermatographism	NO
Patient 16	Ovarian	F	58	Paclitaxel	3	First	Low back pain, respiratory and cardiovascular with hypotension and desaturation (anaphylaxis)	Rash penicillin and cephalexin	ID + 1st dilution	6.2
Patient 17	Breast	F	34	Docetaxel	3	First/second	Flushing and respiratory in 1st infusion; 2nd infusion with tong oedema, convulsion, respirator—anaphylaxis—epinephrine 1 x	NO	ID + 1st dilution	NO

**Table 2:** General characteristics of healthy control group.

**Control**	**Type of tumor**	**Sex**	**Age**	**Drug**	**Grade of Initial reaction**	**Lifetime exposure**	**Symptoms**	**History of atopic disease/HR**	**Cutaneous tests**	**Tryptase**
Control 18	N/A	F	32	N/A	N/A	N/A	N/A	No	N/A	N/A
Control 19	N/A	F	48	N/A	N/A	N/A	N/A	Asthma + Allergic Rhinitis	N/A	N/A
Control 20	N/A	F	51	N/A	N/A	N/A	N/A	No	N/A	N/A
Control 21	N/A	M	35	N/A	N/A	N/A	N/A	No	N/A	N/A
Control 22	N/A	M	42	N/A	N/A	N/A	N/A	Allergic Rhinitis	N/A	N/A
Control 23	N/A	F	26	N/A	N/A	N/A	N/A	Asthma + Allergic Rhinitis	N/A	N/A
Control 24	N/A	F	27	N/A	N/A	N/A	N/A	No	N/A	N/A
Control 25	N/A	F	56	N/A	N/A	N/A	N/A	No	N/A	N/A

Regarding the type of neoplasms, ovarian cancer was the most prevalent (46%), followed by breast cancer (26%) and uterine cancer (13%). Paclitaxel was the chemotherapeutic agent used in 13 patients (87%), while two patients received docetaxel (13%). All reactions occurred in the first or second infusion, 10 (66%) in the first and one in the second (6%). In the remaining cases (26%), patients had a mild reaction in the first infusion and tolerated the reintroduction of medication but progressed with a severe reaction in the next exposures. The serum tryptase value at the time of the initial reaction was recovered from the medical records and only five patients had tryptase collected at the time of initial reaction, being in all cases below the normal value of 11.4 mg/ml, none of them had register of the baseline tryptase values to compare vs. initial reaction time values. Two of the 15 patients could not undergo the skin testing because they had contraindications, one had a false negative result due to premedication with antihistamine and one and false positive results due to dermatographism (Table 1). No patient had a positive skin test for taxanes in the puncture test, and most were positive at the lowest dilution of the intradermal test, at a concentration of 0.01 mg/ml for paclitaxel.

### Basophil Activation Test

In total, 15 BATs were performed in the patient group and eight in the control group. The concentration that showed the highest positivity was 0.1 mg/ml for paclitaxel (1:10 of the initial 1:1 dilution of 1 mg/ml) and 0.04 mg/ml for docetaxel (1:10 of the initial dilution 1:1 of 0.4 mg/ml). The stimulation index was calculated by dividing the MFI of CD63 and CD203c after paclitaxel or docetaxel stimulation by the MFI CD63 and CD203c after PBS stimulation for each of the markers. Table 3 shows the basophil stimulation index (SI)from the cell-surface expression of CD63 and CD203c in patients and controls after paclitaxel or docetaxel stimulation at a 1:10 and 1:100 dilutions vs. PBS (negative control).

**Table 3:** Stimulation index from the CD63 and CD203c expression of patients and healthy controls after stimulation with paclitaxel or docetaxel vs. PBS (negative control).

	**CD203c**		**CD63**	
	**Taxol 1:10**	**Taxol 1:100**	**Taxol 1:10**	**Taxol 1:100**
Patient 2	2.82	1.52	1.85	1.36
Patient 3	4.14	4.14	1.97	2.34
Patient 4	0.57	0.76	0.98	1.06
Patient 5	0.72	0.77	0.73	0.78
Patient 6	2.94	10.63	1.26	6.95
Patient 7	1.28	0.89	1.61	0.93
Patient 9	1.15	0.82	1.49	0.90
Patient 10	0.97	1.38	0.67	1.19
Patient 11	1.31	0.79	1.9	0.84
Patient 12	1.22	1.59	1.18	1.80
Patient 13	3.12	2.50	1.18	1.11
Patient 14	2.45	1.03	1.71	0.60
Patient 15	1.77	1.19	4.35	1.41
Patient 16	2.53	2.19	2.96	1.95
Control 18	0.92	0.90	1.14	1.29
Control 19	1.17	1.09	1.03	0.96
Control 21	3.2	1.50	1.4	1.03
Control 22	1.11	0.56	1.87	1.04
Control 23	0.95	0.99	1.31	1.29
Control 24	1.16	1.15	1.03	1.03
Control 25	0.88	0.87	1.06	1.18
Control 26	1.27	0.67	1.73	0.88
Control	**Taxotere 1:10 (0.4)**	**Taxotere 1:100 (0.04)**	**Taxotere 1:10 (0.4)**	**Taxotere 1:100 (0.04)**
Control 17	1.05	1.00	1.27	1.21

*Cut-off for CD203c 1.3 and for CD63 1.8 (patients # 1 and # 8 were excluded due to initial reaction grade 1)*.

**Figure 1:**
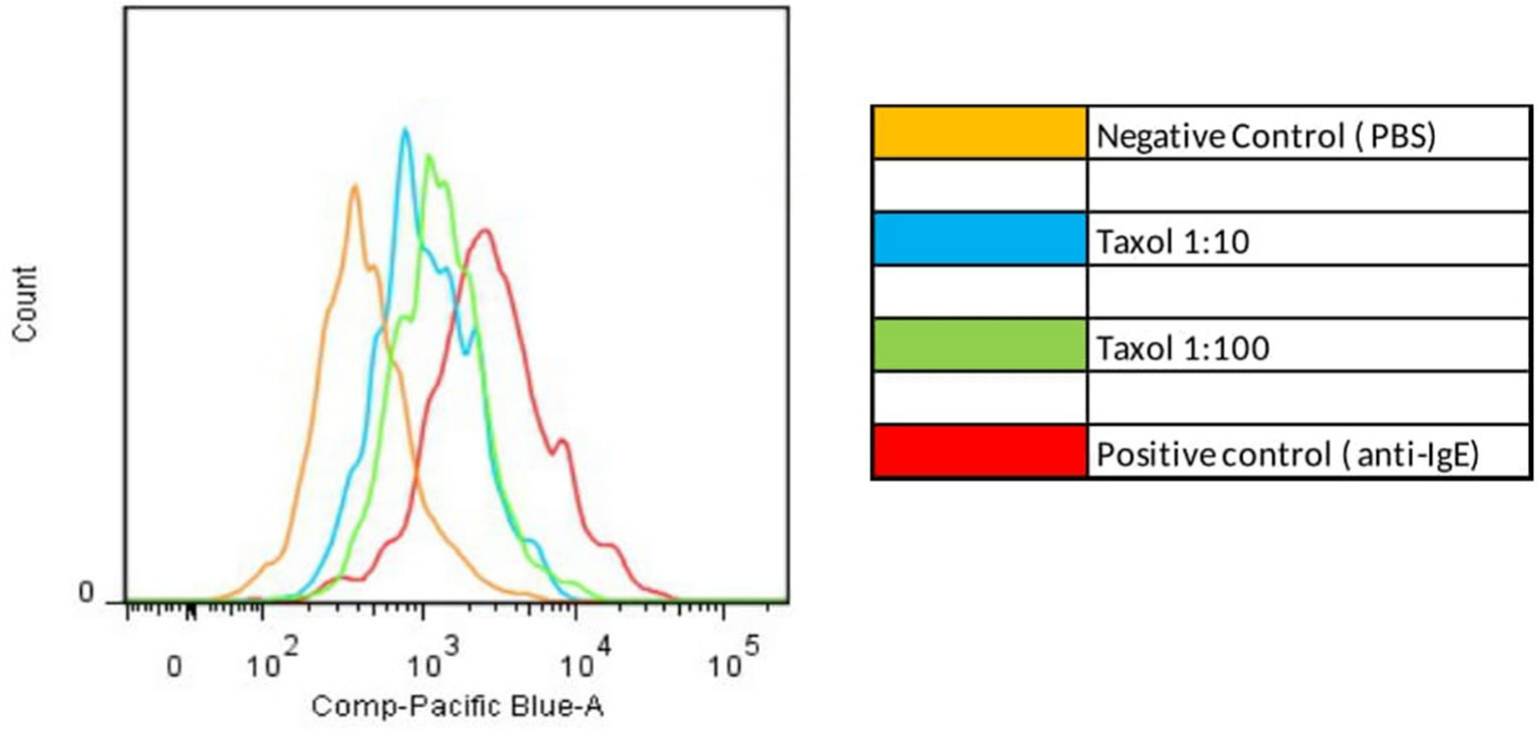
Positive Basophil Activation test. Increased MFI after basophil stimulation with paclitaxel compared with negative control (PBS).

The cutoff value for a positive BAT (threshold) was calculated by drawing the ROC curve for the stimulation index, comparing values of the taxane stimulation indices vs. PBS. The results chosen for this analysis were the SI after stimulation with the dilution of 1:10 for both paclitaxel and docetaxel, because this was the concentration that showed the higher MFI values for the majority of samples analyzed. The ideal cutoff point for the test is where the combination of sensitivity and specificity reaches the maximum point of the curve. For CD63, the cutoff point is 1.8, and for CD203 is 1.3. Figure 1 shows the ROC curve for the stimulation index of CD3 expression.

**Figure 2:**
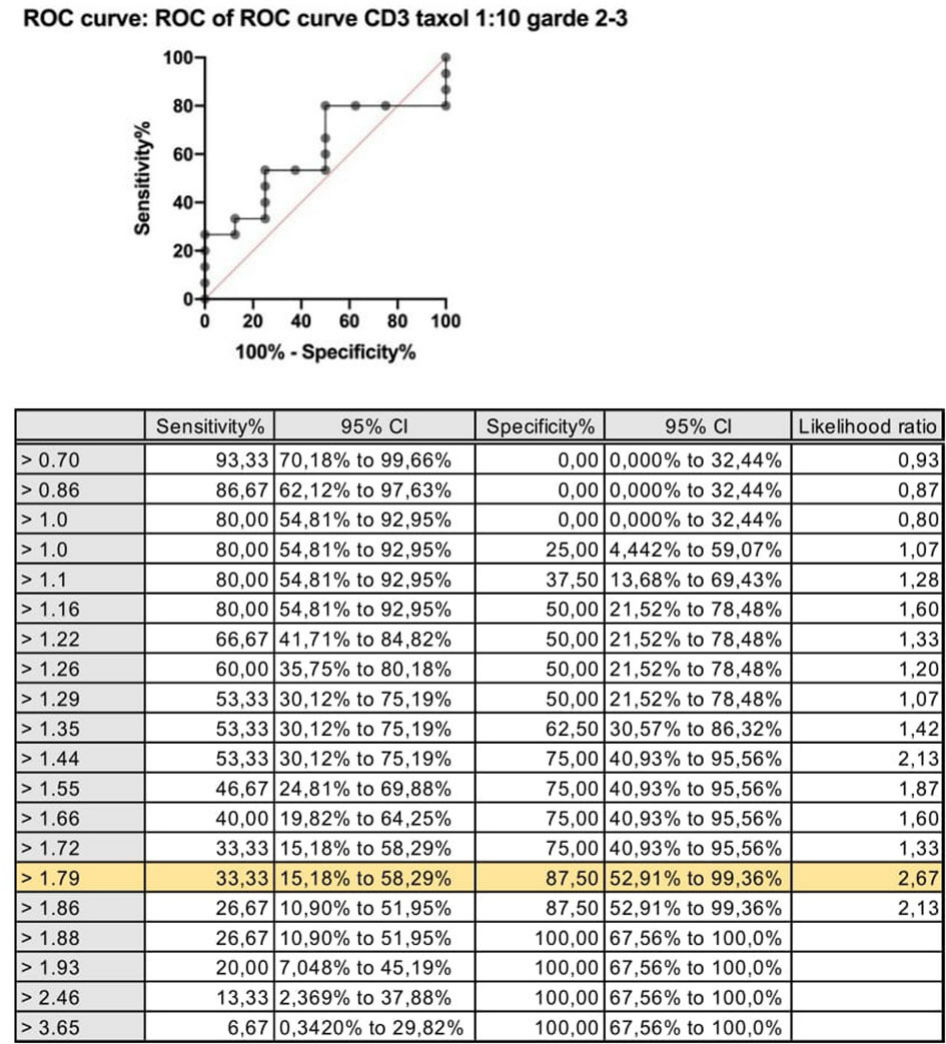
ROC curve: stimulation index for CD63 expression after stimulation with paclitaxel 1:10 vs. saline CD63: Threshold > 1.8; sensitivity 33% and specificity 88%.

**Figure 3:**
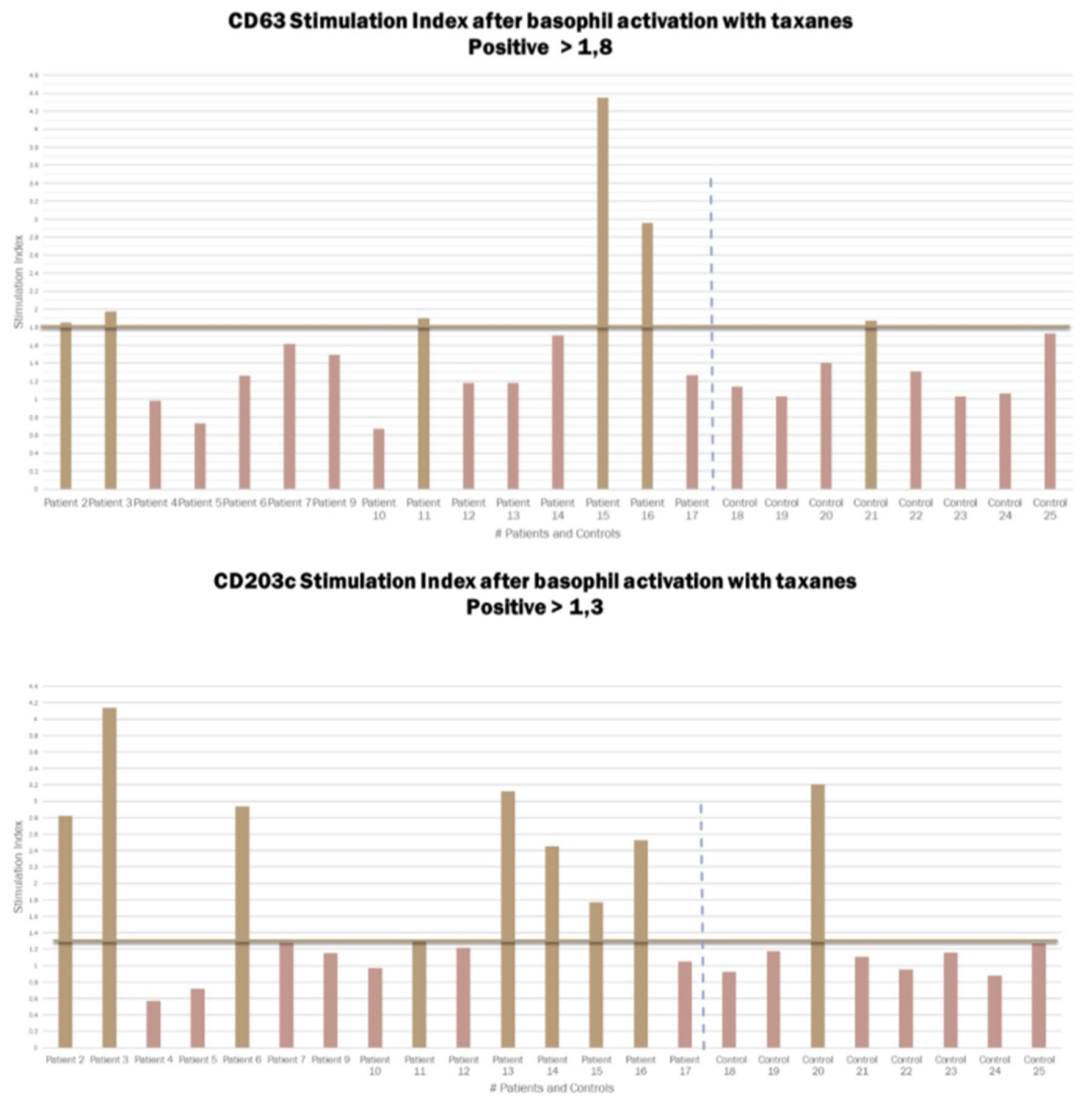
Graphic representation of the Stimulation index from the CD63 and CD203c expression in patients and controls after basophil activation with taxanes. Cut-off for CD203c 1.3 and for CD63 1.8.

BAT was positive in eight of the 15 patients tested (**Figure 3**). There was an increase in CD203c expression in 8/15 (53%) patients and CD63 in 5/15 (40%) patients. The sensitivity for CD203c was 53%, and the specificity was 87% (area under the curve 0.66; *p* = 0.19%). For CD63, these rates were 33 and 87%, respectively (area under the curve 0.6; *p* = 0.4). All patients with increased CD63 expression had increased CD203c expression. Figure 2 shows an example of a BAT with a positive result, expressed by MFI, showing the increase of CD3 MFI after stimulation with paclitaxel vs. stimulation with PBS. The higher MFI is represented by stimulation with anti-IgE, as expected, for positive control. Two of the eight individuals tested in the control group had a positive BAT (25%): one for CD63 and one for CD203c.

We performed a subgroup analysis excluding patients who did not realized the skin test due to contraindications and patients who a had false positive or a false negative result (Table 1). The objective of this sub-group analysis was to evaluate a sample containing only patients with positive skin tests, and IgE mediated endotype confirmed (11 patients). The sensitivity and specificity of BAT were not significantly changed. In this group of patients, CD203c was positive in six patients (sensitivity of 54.5% and specificity of 87.5%), and CD63 was positive in five patients (sensitivity of 45% and specificity of 75%).

## Discussion

We analyzed the application and relevance of BAT as a tool in the diagnosis of immediate hypersensitivity reactions to taxanes. We present the analysis of the BAT results of 15 patients diagnosed with anaphylaxis to taxanes, and in eight healthy volunteers, which is the largest sample of patients who underwent BAT for taxanes.

Female patients diagnosed with ovarian cancer predominated in our sample, representing almost 50% of all cases. Female sex is a known risk factor for immediate hypersensitivity reactions in general (36), but our sample did not have enough participants for us to conclude that female sex was an independent risk factor for anaphylactic reactions to taxanes. Neither data to conclude that ovarian cancer is a risk factor for these reactions. History of atopic disease or hypersensitivity reactions were present in 60% of patients and in 60% of the control group. In the present study, all the initial reactions occurred after the first or second infusion, similar to what has been reported in other studies.

The mechanisms responsible for the immediate hypersensitivity reactions to taxanes are not yet fully understood. The fact that the initial reaction happens mostly associated with the first and second infusion, without prior exposure and with low opportunity for prior sensitization to the drug, raised initial hypothesis of non-IgE-mediated pathways, such as complement activation with release of anaphylatoxins, or direct activation of mast cells and basophils (7).

Paclitaxel and docetaxel are both insoluble compounds. To make the drug formulation suitable for intravenous injection, Paclitaxel is formulated in Cremophor EL (polyoxyethylated castor oil), a surfactant, solubilizer and emulsifying agent and Docetaxel, in polysorbate 80 (Tween 80), another surfactant, solubilizer and emulsifying agent, and further diluted in an ethanol/water (7). Studies with those solvents showed that, *in vitro*, the Cremophor EL alone triggers complement activation at the same rate as Cremophor EL combined with paclitaxel, suggesting that Cremophor EL is responsible for this effect (18). Likewise, docetaxel with polysorbate 80 or polysorbate 80 alone has been shown to cause complement activation *in vitro* (19). Based on these findings, the solvents Cremophor EL and polysorbate 80 could be responsible for triggering hypersensitivity reactions, but this hypothesis has not been confirmed *in vivo*.

The possibility of an IgE-mediated mechanism was raised by Prieto García and Pineda de la Losa. The authors described the first case in the literature of a grade 3 immediate hypersensitivity reaction to paclitaxel with skin testing positive for paclitaxel and negative for the solvent (20). After this, other groups began to study the positivity of skin tests for taxane hypersensitivity reactions. Pagani et al. evaluated the role of immediate-reading skin testing for taxanes in a multicenter study, in which 84 patients with hypersensitivity reactions to taxanes were assessed. Patients with grade 3 hypersensitivity reactions more often had positive skin tests than patients with grade 1–2 reactions (28.6 vs. 10.7%; *p* = 0.038). Patients with positive tests had a higher frequency of skin symptoms. All patients in the control group had negative skin tests, characterizing the low sensitivity (8.7–24.6%) but high specificity (100%) of the test (10). Based on the data described above, IgE-mediated reactions are more likely among patients with more severe and reactions with skin manifestations (9, 10).

We also know that BAT results vary according to the drug class studied and the mechanism of the immediate HR involved (21). BAT has shown promising results in IgE-mediated immediate HR (ex. selective reactors to pyrazolone, neuromuscular blockers, beta-lactams, and platinum compounds), and lower sensitivity for drugs related to non-IgE mechanisms (ex. Non-steroidal anti-inflammatory drugs and quinolones) (21).

In our sample, we selected 15 patients who had anaphylaxis to taxanes with cutaneous manifestations, who had a higher probability of having had an IgE-mediated reaction and positive BAT. Nevertheless, BAT was positive in only 8/15 patients tested (53%). There was an increase in CD203c expression in 8/15 (53%) patients and CD63 in 5/15 (40%) patients.

Of the 15 patients, 11 had positive skin tests. We ran a sub-group analysis, to evaluate if patients with positive skin test have a different outcome. In this analysis CD203c was positive in six (sensitivity of 54.5% and specificity of 87.5%) and CD63 was positive in five (sensitivity of 45% and specificity of 75%). The sensitivity of BAT in this subgroup of patients with a higher probability of IgE-mediated hypersensitivity was not significantly higher than that in the full group. Increased expression of CD203c was more frequent than of CD63 in patients with positive BAT, and the sensitivity of this biomarker was greater in patients with positive skin tests, i.e., patients with IgE-mediated endotypes.

Two patients in the control group of healthy volunteers who had never been exposed to taxanes also showed positive BATs to taxanes, decreasing the specificity of the test. One hypothesis to explain this fact is that the sensitivity to taxanes could be developed without exposure to the drug, because of cross-reactivity with other antigens. Paclitaxel and the precursors for docetaxel (baccatin III and 3-deacetylbaccatin III) have been isolated from different species of yew trees and from different parts of the plant including its pollen. Paclitaxel-specific IgG were found in the serum of 8.8 % of 63 healthy blood donors from Belgium (where T. baccata is widespread) despite having never been exposed to the drug. Among atopic blood donors (defined as sensitive to Betula species), this percentage increased to 21.4 %. In contrast, paclitaxel specific IgGs were not detected in 50 healthy blood donors from the southern hemisphere (where yew trees do not grow) (22). There is evidence in some patients reactive to protamine, that drug specific IgGs (in combination with complement) can mediate immediate HSRs. Although this mechanism has not been explored in taxane HSRs, it may turn out to be relevant. Another possibility is that paclitaxel specific IgE are produced in patients exposed to yew tree pollen. The controls who presented positive BAT for taxanes could be sensitized even without having previous exposure to the drug. The solvents could also trigger complement activation in healthy subjects and thereby to cause immediate HSRs through anaphylatoxin production and histamine release could also be triggered through a direct but undefined effect of paclitaxel and docetaxel on basophil/mast cells in health subjects.

## Conclusion

BAT sensitivity for taxanes is 53% and specificity 87%. The expression of CD203c was more frequent than of CD63 in the patients with positive BAT. We conclude that BAT for the diagnosis of anaphylaxis to taxanes can be a useful diagnostic tool for patients with specific phenotypes and endotypes, especially with severe immediate HR. For the positive patients, this new diagnostic toll will avoid to skip-first line therapies, and desensitization can be indicated.

## Data Availability Statement

The original contributions presented in the study are included in the article/supplementary material, further inquiries can be directed to the corresponding author/s.

## Ethics Statement

The project was submitted to and approved by the research Ethics Committees Partners Institutional Review Board (protocols 13–288 and 2012P002275) and CAPPesq of the Hospital das Clínicas (Brazil Platform: CAAE: 22701913.6.0000.0068). The patients/participants provided their written informed consent to participate in this study.

## Author Contributions

All authors listed have made a substantial, direct, and intellectual contribution to the work and approved it for publication.

## Conflict of Interest

The authors declare that the research was conducted in the absence of any commercial or financial relationships that could be construed as a potential conflict of interest.

## Publisher's Note

All claims expressed in this article are solely those of the authors and do not necessarily represent those of their affiliated organizations, or those of the publisher, the editors and the reviewers. Any product that may be evaluated in this article, or claim that may be made by its manufacturer, is not guaranteed or endorsed by the publisher.

## References

[1] Castells MC Tennant NM Sloane DE Hsu FI Barrett NA Hong DI. Hypersensitivity reactions to chemotherapy: outcomes and safety of rapid desensitization in 413 cases. J Allergy Clin Immunol. (2008) 122:574–80. 10.1016/j.jaci.2008.02.044 18502492

[2] Castells MC. Hypersensitivity to antineoplastic agents. Curr Pharm Des. (2008) 14:2892–901. 10.2174/138161208786369803 18991707

[3] INCA B. *Estimate/2020 - Cancer Incidence in Brazil*. COORDENAÇÃO DE ENSINO. Serviço de Educação e Informação Técnico-Cient#x000ED;fica ed. Rio de Janeiro: Brazil (2019). p. 120.

[4] Markman M Kennedy A Webster K Kulp B Peterson G Belinson J. Paclitaxel-associated hypersensitivity reactions: experience of the gynecologic oncology program of the Cleveland Clinic Cancer Center. J Clin Oncol. (2000) 18:102–5. 10.1200/JCO.2000.18.1.102 10623699

[5] Weiss RB Donehower RC Wiernik PH Ohnuma T Gralla RJ Trump DL. Hypersensitivity reactions from taxol. J Clin Oncol. (1990) 8:1263–8. 10.1200/JCO.1990.8.7.1263 1972736

[6] Picard M Matulonis UA Castells M. Chemotherapy hypersensitivity reactions in ovarian cancer. J Natl Compr Canc Netw. (2014) 12:389–402. 10.6004/jnccn.2014.0040 24616544

[7] Picard M Castells MC. Re-visiting hypersensitivity reactions to taxanes: a comprehensive review. Clin Rev Allergy Immunol. (2015) 49:177–91. 10.1007/s12016-014-8416-0 24740483

[8] Pagani M Bavbek S Alvarez-Cuesta E Berna Dursun A Bonadonna P Castells M. Hypersensitivity reactions to chemotherapy: an EAACI Position Paper. Allergy. (2022) 77:388–403. 10.1111/all.15113 34587281

[9] Picard M Pur L Caiado J Giavina-Bianchi P Galvão VR Berlin ST. Risk stratification and skin testing to guide re-exposure in taxane-induced hypersensitivity reactions. J Allergy Clin Immunol. (2016) 137:1154–64.e12. 10.1016/j.jaci.2015.10.039 26725998

[10] Pagani M Bavbek S Dursun AB Bonadonna P Caralli M Cernadas J. Role of skin tests in the diagnosis of immediate hypersensitivity reactions to taxanes: results of a multicenter study. J Allergy Clin Immunol Pract. (2019) 7:990–7. 10.1016/j.jaip.2018.09.018 30292919

[11] Kleine-Tebbe J Erdmann S Knol EF MacGlashan DW Poulsen LK Gibbs BF. Diagnostic tests based on human basophils: potentials, pitfalls and perspectives. Int Arch Allergy Immunol. (2006) 141:79–90. 10.1159/000094495 16837789

[12] Bühring HJ Seiffert M Giesert C Marxer A Kanz L Valent P. The basophil activation marker defined by antibody 97A6 is identical to the ectonucleotide pyrophosphatase/phosphodiesterase 3. Blood. (2001) 97:3303–5. 10.1182/blood.V97.10.3303 11342463

[13] Bühring HJ Simmons PJ Pudney M Müller R Jarrossay D van Agthoven A. The monoclonal antibody 97A6 defines a novel surface antigen expressed on human basophils and their multipotent and unipotent progenitors. Blood. (1999) 94:2343–56. 10498606

[14] Caiado J Picard M. Diagnostic tools for hypersensitivity to platinum drugs and taxanes: skin testing, specific IgE, and mast cell/basophil mediators. Curr Allergy Asthma Rep. (2014) 14:451. 10.1007/s11882-014-0451-7 24951237

[15] Giavina-Bianchi P Galvao VR Picard M Caiado J Castells MC. Basophil activation test is a relevant biomarker of the outcome of rapid desensitization in platinum compounds-allergy. J Allergy Clin Immunol Pract. (2017) 5:728–36. 10.1016/j.jaip.2016.11.006 28034549

[16] Bonamichi-Santos R Castells M. Diagnoses and management of drug hypersensitivity and anaphylaxis in cancer and chronic inflammatory diseases: reactions to taxanes and monoclonal antibodies. Clin Rev Allergy Immunol. (2018) 54:375–85. 10.1007/s12016-016-8556-5 27277133

[17] Brown SG. Clinical features and severity grading of anaphylaxis. J Allergy Clin Immunol. (2004) 114:371–6. 10.1016/j.jaci.2004.04.029 15316518

[18] Szebeni J Muggia FM Alving CR. Complement activation by Cremophor EL as a possible contributor to hypersensitivity to paclitaxel: an *in vitro* study. J Natl Cancer Inst. (1998) 90:300–6. 10.1093/jnci/90.4.300 9486816

[19] Weiszhár Z Czúcz J Révész C Rosivall L Szebeni J Rozsnyay Z. Complement activation by polyethoxylated pharmaceutical surfactants: cremophor-EL, Tween-80 and Tween-20. Eur J Pharm Sci. (2012) 45:492–8. 10.1016/j.ejps.2011.09.016 21963457

[20] Prieto García A Pineda de la Losa F. Immunoglobulin E-mediated severe anaphylaxis to paclitaxel. J Investig Allergol Clin Immunol. (2010) 20:170–6. 20461974

[21] Campos L Galvão VR Kalil J Castells M Giavina-Bianchi P. BAT in the diagnosis of drug allergy: a novel tool in clinical daily practice? Curr Allergy Asthma Rep. (2019) 19:20. 10.1007/s11882-019-0852-8 30859323

[22] Vanhaelen M Duchateau J Vanhaelen-Fastré R Jaziri M. Taxanes in Taxus baccata pollen: cardiotoxicity and/or allergenicity? Planta Med. (2002) 68:36–40. 10.1055/s-2002-19865 11842324

